# Incidental finding of jejunal duplication cyst during laparotomy: A case report

**DOI:** 10.1016/j.amsu.2022.103971

**Published:** 2022-06-08

**Authors:** Yassine El Berni, Aziz Moufakkir, Mounir Bouali, Abdelilah El Bakouri, Khalid El Hattabi, Fatimazahra Bensardi, Abdelaziz Fadil

**Affiliations:** a Faculty of Medicine and Pharmacy, Hassan II University of Casablanca, Casablanca, Morocco; b Department of of Visceral Surgical Emergency, CHU Ibn Rochd, Casablanca, Morocco

**Keywords:** Jejunal duplication cyst, Congenital anomalies, Abdominal cystic mass, Case report

## Abstract

**Introduction:**

and importance: Duplications, which rare anomalies of the gastroin-testinal tract, may be located in any part of the gastro-intestinal system from the oral cavity to the anus. The aim of this paper is to present and discuss a case of an asymptomatic jejunal duplication cyst associated with adhesive small bowel obstruction in an elderly lady.

**Case presentation:**

A 70-year-old female, presented to the emergency room with a history of recurrent abdominal, non-bilious vomiting, and abdominal distension for 5 days with no passage of stool and flatus for 3 days. Abdominal Computed tomography scan showed dilated fluid-filled small bowel loops with abrupt transition to collapsed small bowel associated with a focal kink and narrowing of the lumen. The patient was rushed for emergency laparotomy for diagnosis of adhesive small bowel obstruction. To our surprise, jejunal duplication cysts were found.

**Clinical discussion:**

the intestinal duplication cysts are rare congenital anomalies. The clinical presentation is variable and depends on the site and the related complications. Surgical resection is deemed appropriate management due to known complications like obstruction, hemorrhage, perforation, and malignant degeneration.

**Conclusion:**

It's important to include intestinal duplication in the differential diagnosis of acute abdomen.

## Highlights

• Duplications of the gastrointestinal tract are rare congenital anomalies.
• Duplications are located on the mesenteric border of the associated native bowel.
• The resection of the involved bowel is the treatment associated with the best long-term outcomes.

## 1 Introduction

Duplications, which rare anomalies of the gastro-intestinal tract, may be located in any part of the gastrointestinal system from the oral cavity to the anus [1]. Duplications are most frequently single. They are located on the mesenteric border of the associated native bowel and vary in size and shape: cystic in 80% of cases and tubular in the remaining 20%, with or without other congenital anomalies [2].

Enteric duplication cysts is most frequently observed in the terminal part of the ileum [3]. More than 80% of the cases present before the age of 2 years as an acute abdomen or bowel obstruction, but many duplications remain silent unless complications occur, and therefore may not be diagnosed until adulthood [2]. Complications include volvulus, bleeding, and, rarely, malignant degeneration [4].

We present a case of an asymptomatic jejunal duplication cyst associated with adhesive small bowel obstruction in an elderly lady. This work has been reported in line with the scare 2020 criteria [5].

## 2 Case presentation

A 70-year-old female, with a prior surgical history of appendicular peritonitis 23 years back, presented to the emergency room with a history of recurrent abdominal, non-bilious vomiting, and abdominal distension for 5 days with no passage of stool and flatus for 3 days. There was no history of fever, jaundice, or weight loss. The patient had no medical history and no history of alcohol or drug abuse. The Patient had no significant family history.

On general examination, she was afebrile with a Blood Pressure of 120/70 mm of Hg, pulse rate of 60 beats per minute, respiratory rate of 18 breaths per minute, and Sp0 2 of 97% in the room air. On local examination, the abdomen was distended, diffusely tender, with an absence of bowel sounds.

Laboratory and biochemical investigations were within normal limits. Plain X-ray finding of dilated proximal small bowel with multiple air-fluid levels and CT scan showed dilated fluid-filled small bowel loops with abrupt transition to collapsed small bowel associated with a focal kink and narrowing of the lumen.

**Fig. 1:**
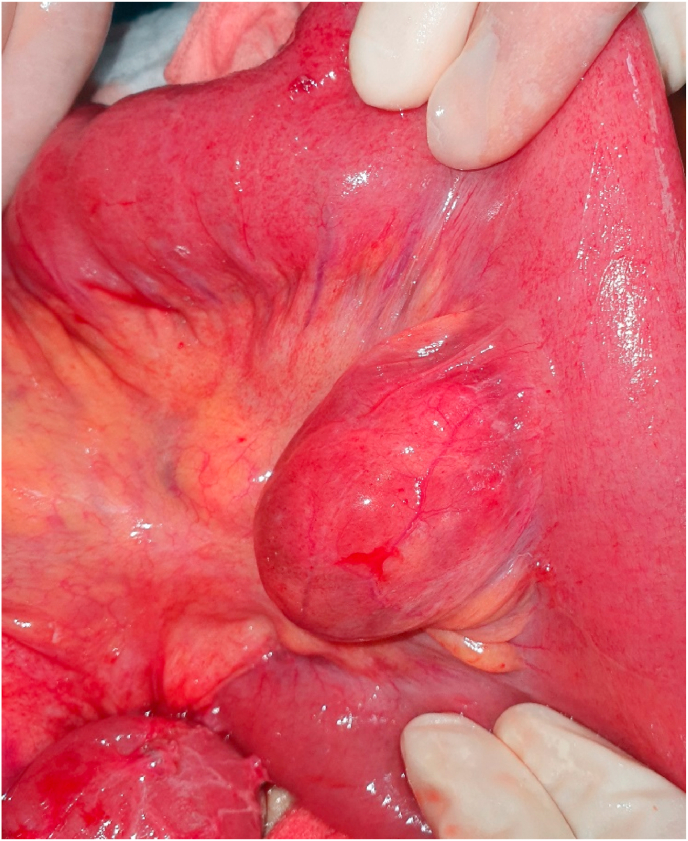
Intraoperative image of jejunal duplication cyst.

**Fig. 2:**
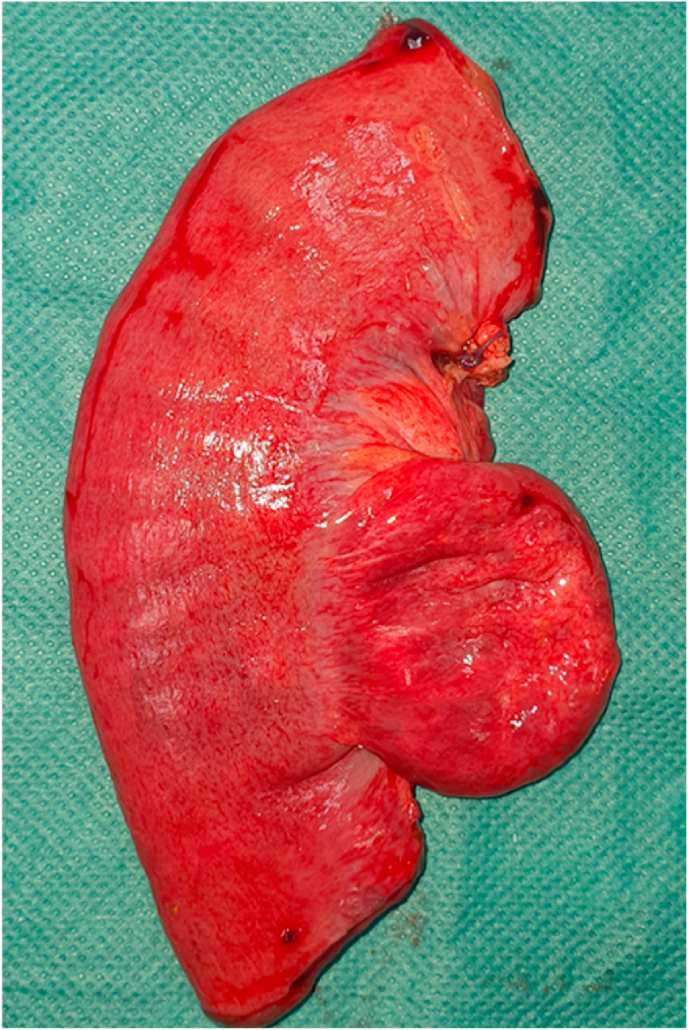
Resected segment of jejunum with cystic duplication.

The patient was rushed for emergency laparotomy for diagnosis of adhesive small bowel obstruction. During surgery, multiple adhesions between loops of the small bowel, with dilation of proximal bowel were found. To our surprise, Enteric Duplication cysts, communicating with the lumen, in the jejunum, 10 cm distally from duodeno-jejunal flexure were found (Fig. 1). An extensive search did not show duplication in the small and large bowel. Adhesiolysis was done and resection of jejunal duplication cysts was performed with end-to-end anastomosis (Fig. 2). The Final histopathology report showed a duplication cyst lined with jejunal mucosa without any ectopic tissue.

The patient was started on enteral feeding on postoperative day 5 and discharged without any complications. On follow-up after 2 months, she was doing well and was planned for yearly follow.

## 3 Discussion

Enteric Duplication cysts are rare congenital malformations that consist of cystic formation in communication with the native gastro-intestinal tract, sharing a common muscular wall and blood supply [6]. The etiology of gastrointestinal duplications has not been well determined, while several theories have been proposed: partial twining, split notochord, environmental factors (trauma or hypoxia), embryonic diverticula, and recanalization defects [7].

Duplication cysts have a smooth muscle layer in the cyst wall, and the cyst is lined with the mucosa of the adjacent alimentary tract, which may contain ectopic digestive tract tissue such as gastric mucosa or pancreatic tissue [8,9]. In our case, the duplication was detected in the jejunum, where it is less commonly seen, and was a cystic duplication, which is the most common type.

The clinical presentation is extremely variable, depending on localization, shape, size, and type of mucosa, most of the duplications become symptomatic before age 2, and a few may remain asymptomatic as seen in our patient [4]. In our case, clinical symptoms were abdominal pain, nausea, and vomiting, but these symptoms were related to the accompanying adhesive small bowel obstruction since the jejunal duplication was non-complicated.

Surgical treatment is needed in both symptomatic patients and in asymptomatic with a secondary diagnosis for the high prevalence of complications like enteric obstruction, bleeding, volvulus, and rare malignant transformation in adult age [10,11].

## 4 Conclusion

We highlight the rare occurrence of terminal jejunal duplication cyst in the elderly. Surgical treatment is necessary due to the probable malignant transformation of duplication cysts. It is important to include intestinal duplication in the differential diagnosis of acute abdomen.

## Provenance and peer review

Not commissioned, externally peer-reviewed.

## Sources of funding

None.

## Ethical approval

I declare on my honor that the ethical approval has been exempted by my establishment.

## Consent

Written informed consent for publication of their clinical details and/or clinical images was obtained from the patient. A copy of the written consent is available for review by the Editor-in-Chief of this journal on request.

## Research registration

1. Name of the registry: None
2. Unique Identifying number or registration ID: None
3. Hyperlink to your specific registration (must be publicly accessible and will be checked):

## Guarantor

Yassine El Berni.

## Declaration of competing interest

The authors declare having no conflicts of interest for this article.
